# Methods to Assess Social Comparison Processes Within Persons in Daily Life: A Scoping Review

**DOI:** 10.3389/fpsyg.2019.02909

**Published:** 2020-01-22

**Authors:** Danielle Arigo, Jacqueline A. Mogle, Megan M. Brown, Kristen Pasko, Laura Travers, Logan Sweeder, Joshua M. Smyth

**Affiliations:** ^1^Department of Psychology, Rowan University, Glassboro, NJ, United States; ^2^Prevention Research Center, Pennsylvania State University, State College, PA, United States; ^3^Department of Nursing, Pennsylvania State University, State College, PA, United States; ^4^Departments of Biobehavioral Health and Medicine, Pennsylvania State University, State College, PA, United States

**Keywords:** social comparison, within-person, ecological momentary assessment, daily diary, intensive longitudinal data, ambulatory assessment, social influence

## Abstract

Self-evaluations relative to others (i.e., *social comparisons*) have well-established implications for health and well-being, and are typically assessed via global, retrospective self-report. Yet, comparison is inherently a dynamic, within-person process; comparisons occur at different times, on a range of dimensions, with consequences that can vary by context. Global, retrospective assessment forces aggregation across contexts and reduces ecological validity, limiting its utility for informing a nuanced understanding of comparisons in daily life. Research across social and clinical psychology has implemented methods to assess comparisons naturalistically, involving intensive, repeated assessments of comparison occurrence, characteristics, and consequences in everyday life (via ecological momentary assessment or daily diaries). Although promising, this work to date lacks an overarching conceptual framework for guiding decisions about assessment design and implementation. To address this gap, the aims of this scoping review were: (1) to summarize available literature on within-person naturalistic assessment of social comparison, and (2) to provide a set of key considerations to inform future social comparison research using within-person naturalistic assessment. Searches in PubMed, PsycInfo, and CINAHL identified relevant articles published before June 2019. Articles were included if they described at least 3 comparison assessments within each participant, taken in the natural environment, and spaced no more than ~24 h apart (i.e., repeated momentary or daily assessment). In articles meeting these criteria (33 unique studies across 36 published papers), we summarized aspects of the comparison assessment, including recording methods, direction (e.g., upward, downward), target (e.g., friend, stranger), and dimension (e.g., status, appearance). Most studies assessed appearance comparisons (vs. other comparison dimensions) and collected information in response to signals (rather than initiated by participants). However, there was considerable heterogeneity in the number of assessments, assessment periods, recording modalities, and comparison predictors and outcomes assessed. Findings broadly establish heterogeneity in the aspects of comparison considered critical for within-person naturalistic assessment. We describe key decision points for future work to help advance within-person naturalistic assessment methods and improve the utility of such approaches to inform research, theory, and intervention.

To date, more than six decades of research has demonstrated that self-evaluations relative to others (i.e., *social comparisons*) have important implications for well-being and health (Buunk and Gibbons, 2007; Gerber et al., 2018). For example, a range of research designs and assessment methods has shown that comparisons can influence intrapsychic states such as affect and attitudes (Myers, 1978; Buunk et al., 1990), satisfaction with one's current status (Major and Testa, 1989), and perceptions of risk for negative outcomes (Klein and Weinstein, 1997). Social comparisons also contribute to mental health conditions such as depression (Swallow and Kuiper, 1988) and to physical health outcomes such as smoking (Gerrard et al., 2005), weight loss (Leahey et al., 2011b), and chronic illness self-care (Arigo et al., 2015), and comparison is recognized as a key mechanism underlying health behavior change (Olander et al., 2013). Consequently, there is considerable interest in effectively harnessing comparison processes to promote healthy behavior and self-concept.

To achieve this goal, however, it will be critical to understand for whom, when, and under what circumstances social comparison is most likely to confer benefits. Answering these questions requires applying distinct research methods that capture comparisons at different levels of influence. At the person level, considerable evidence shows that people differ in their tendency to compare themselves with others (e.g., *social comparison orientation* [SCO]; Gibbons and Buunk, 1999; O'Brien et al., 2009), and recognizing this between-person difference has generated useful insights. For example, this work has demonstrated that those who have a stronger (vs. weaker) SCO respond more (vs. less) intensely to comparison opportunities (Vogel et al., 2015). Whether this is beneficial is unclear, however; SCO is positively associated with neuroticism, depression, and negative affect (Gibbons and Buunk, 1999), but also is positively associated with empathy for others (Buunk and Gibbons, 2006) and engagement in physical activity (Luszczynska et al., 2004; Arigo and Butryn, 2019).

How might social comparison processes relate to both positive and negative outcomes? It seems that not all instances of comparison are created equal; their effects depend on a variety of factors such as type of comparison target, comparison dimension and direction, and the comparer's perceived similarly to the comparison target (see Table 1). These contextual factors differ between instances of comparison and thus vary within the same person over brief periods of time. In fact, Gibbons and Buunk (1999) note that their measure of SCO has suboptimal temporal stability for a measure of individual differences (i.e., 0.60), in part, because comparison activity is expected to vary with contextual changes. Similarly, Van der Zee et al. (2000) measure of comparison response shows that the same individuals report experiencing both positive and negative affect across instances of comparison, suggesting within-person variability in comparison experiences over time and context.

**Table 1:** Features of social comparison commonly described in theoretical and empirical literature.

**Feature**	**Definition**
Type of comparison target	Category of person or relation to the self—e.g., friend (in real life or on social media), family member, work colleague, stranger, celebrity
Comparison dimension	Aspect of the self or behavior being compared to that of others—e.g., income, professional status, ability, appearance, progress toward a goal
Comparison direction	Perception of the target's status relative to the self on the relevant comparison dimension
Upward comparison	Target is perceived to be better off than the self
Lateral comparison	Target is perceived to be at the same level as the self
Downward comparison	Target is perceived to be worse off than the self
Perceived similarity to the target	During or immediately after a comparison, emphasis on similarities with vs. differences from the target on the relevant comparison dimension
Identification	Emphasis or focus on similarities or closeness between the self and the target
Contrast	Emphasis or focus on differences or distance between the self and the target
Comparison mode	Immediate level of interaction with the comparison target—e.g., in person, over the phone, on social media, on television, in a magazine

People make comparisons to friends, family members, work colleagues, strangers, and celebrities, among other types of *targets*, and some targets may be more important in certain contexts than others (Wheeler and Miyake, 1992; Leahey and Crowther, 2008). The *dimension* of comparison refers to what about the self is being compared—for example, income, professional status, ability in a specific arena (e.g., playing an instrument), appearance, or progress toward a goal—and the value of each dimension may depend on both the person and instance of comparison. The comparison *direction* depends on a person's perception of the target's status (in the dimension under comparison) relative to their own. Comparisons to those perceived as better off on a relevant dimension are *upward comparisons*, comparisons to those perceived as worse off on this dimension are *downward comparisons*, and comparisons to those perceived to be about the same on this dimension are *lateral comparisons* (Wills, 1981; Wood et al., 1985).

From work on individual differences in social comparison behavior (using self-report methods), some people are more likely to make comparisons with specific types of targets or on specific dimensions than others. For example, young adults make comparisons to friends more often than to family members (Wheeler and Miyake, 1992), women with (vs. without) elevated body dissatisfaction are more likely to make appearance comparisons (Leahey et al., 2011a), and people with (vs. without) depression or anxiety make more upward comparisons (Butzer and Kuiper, 2006). But studies that use selection methods (Wood, 1996)—i.e., assessing participants' choice of target from a set of multiple options—reveal that people show a range of preferences for specific comparison directions and dimensions when given options, and that their preferences may not be consistent over time (Van der Zee et al., 1998b; Arigo et al., 2015). Thus, in addition to the overall frequency of comparison type, the specific features of a given comparison may matter much more than previously thought.

Understanding whether making social comparisons is associated with positive or negative outcomes for a given individual requires even more nuance. With respect to immediate emotional states/affect, Buunk and Ybema (1997) proposed that people who identify with upward targets (by focusing on similarities with the target) tend to feel inspired by someone else's success, as a similar outcome for the self seems possible, whereas people who contrast themselves against upward targets (by focusing on differences between the self and the target) tend to feel disappointed by the distance between their current and desired states. Conversely, people who identify with downward targets tend to feel anxious in response to apparent confirmation that their situation is or may become dire, whereas people who contrast with downward targets (by focusing on differences) tend to feel satisfaction with their own status, as the target shows them that they could be doing worse (see Buunk and Ybema, 1997).

Importantly, however, all of these immediate affective responses to social comparison—inspiration, disappointment, anxiety, or satisfaction—may motivate behavior change (Castonguay et al., 1998). Whether they lead to positive changes depends not only on who makes the comparison, but on a variety of contextual influences; in addition to comparison direction and the degree of similarly vs. difference the comparer perceives, the outcome of a comparison may depend on the time of day or week, pre-comparison mood state, reason for making this comparison, or the comparer's previous progress toward their goals (Wheeler and Miyake, 1992; Aspinwall and Taylor, 1993; Arigo et al., 2018). Our understanding of these contextual factors (which may vary within the same person over short periods of time) and their roles in the comparison process is limited by the use of between-person research methods (i.e., retrospective self-report and group-based experimental designs), which tend to be most common in social comparison research. These methods force aggregation both within an individual across occasions and contexts as well as across individuals.

For example, in reporting their tendency to engage in social comparison, individuals must retrospect over their experiences with social comparison and provide a single (usually numeric) answer. This answer typically is intended to reflect an individual's perception of the frequency with which they make comparisons or their perception of how strongly they value comparison information (or both), and measures that assess this construct often do not specify a time frame. This answer will be differentially influenced by a number of factors, including recent experiences, intense/salient experiences (Do et al., 2008; Schneider et al., 2011; i.e., peak and end effects), social desirability (Furnham, 1986), stereotypes or pre-existing beliefs (Cavanaugh et al., 1998), and related but separate current states (e.g., current level of negative affect; Robinson and Clore, 2002a,b). The role that each of these factors plays in an individual's internally generated summary score likely differs across individuals, providing a differently weighted aggregation of experiences in a single set of responses that is then combined to compare individuals to one another (Hill et al., 2018).

Further, aggregation likely reduces ecological validity, as it dissociates the experience of social comparison from the real-world contexts where it occurs (Sliwinski et al., 2018). Thus, asking individuals to provide a single response about their comparison behavior that is a summation over multiple contexts could remove meaningful variation in comparisons that occur in response to real world situations and events, and removes temporal sequencing regarding predictors and/or outcomes. Similarly, group-based experimental designs that present individuals with a single target for comparison and capture reactions to the comparison (e.g., Stanton et al., 1999; Derlega et al., 2008), although high on internal validity, reduce the extent to which the comparison reflects the type of target that individual would select or respond to in the real world. These designs provide information about how comparisons function in response to a specifically generated target, but not how the individual goes about choosing or responding to targets in their everyday life. To achieve the goal of promoting healthy outcomes, there is need to better understand how comparisons occur and function in people's daily lives, with greater attention to specific experiences with comparison, and how these dynamic processes unfold within an individual.

In response to this need, a subset of research across social and clinical psychology has begun to assess specific experiences of social comparison that occur in the natural environment to understand the dynamics of social comparison processes. This involves ambulatory assessment of self-reported comparisons in daily life, using repeated assessment of the same participants over short time intervals (e.g., hours or days; vs. single-administration, global self-report; Smyth et al., 2017). Assessments can occur after a set amount of time (i.e., interval-contingent recording, such at the end of each day), in response to a prompt from the research team (i.e., signal-contingent recording, usually with technological assistance) or in response to a participant's recognition that they have made a comparison (i.e., event-contingent recording). A range of terms have been used to describe this general approach, including “experience sampling” (Larson and Csikszentmihalyi, 2014) and “daily diaries” (Gunthert and Wenze, 2012). However, the use of multiple assessments per day is more often called “ecological momentary assessment” (EMA; Smyth and Stone, 2003; Smyth et al., 2017), whereas “daily diary” may refer to recording only once per day. Recording of comparisons and other variables of interest occurs via paper forms, personal digital assistant (PDA), electronic surveys (via links sent by email or text), or standalone smartphone applications.

As comparisons can happen quickly and automatically as well as deliberately (Gilbert et al., 1995), these methods are useful for reducing recall bias and forgetting. Further, these methods can elucidate important *within-person* (i.e., time-sensitive and dynamic) effects that differ from those observed *between-person* (i.e., stable, trait level). For example, consider the relation between exercise and heart rate. At the between-person level, there is a negative relation such that people who exercise more often generally have lower heart rates. But this relation does not hold for the within-person level—when people exercise, their heart rates increase, rather than decrease (cf. Curran and Bauer, 2011). Thus, knowing that people who are high (vs. low) in SCO also exercise more frequently does not mean that we know whether exercise is more or less likely after a comparison happens in the real world. To know the latter, we need intensive assessment of the same person over short time periods to detect within-person, time-sensitive effects.

Intensive assessment approaches to measuring social comparison have been used with the intention to capture the frequency of occurrence, characteristics, and consequences of comparisons, and variability in these aspects of comparison, within an individual at the moment or day levels. Yet, with respect to social comparison, intensive assessment work has moved forward with little coherence across study methods or consensus as to best practices for this approach, and without a framework for guiding decisions about assessment design. Given the nuances of social comparison and the range of methods and parameters that could facilitate intensive assessment, having synthesis of existing studies and specific recommendations for this work could improve the rigor and utility of future studies. In line with these goals, the aims of this scoping review were: (1) to summarize the available literature on intensive assessment of social comparison, regarding the aspects of comparison deemed critical for such assessment (e.g., direction) and specific methods of assessment (e.g., recording method), and (2) to propose a set of key questions to guide decisions about future intensive assessment of social comparison.

## Method

This review followed the PRISMA Extension for Scoping Reviews (PRISMA-ScR; Tricco et al., 2018). A brief description of the protocol for this review is registered with the Open Science Framework (https://osf.io/mbucg/). The research questions for this review were:

1. In which populations and under what circumstances have researchers assessed social comparison processes in the natural environment?
2. What specific assessment procedures, including the number and timing of assessments per day/week and instructions to participants, have been used to capture social comparisons in the natural environment?
3. What characteristics of self-reported social comparisons have been assessed in the natural environment?
4. How frequently do participants report comparisons in the natural environment?
5. What experiences have been assessed as predictors or outcomes of comparisons in the natural environment?

### Article Identification

Inclusion criteria for this review were selected by the first, second, and last authors (DA, JAM, JMS) to focus on intensive, naturalistic assessment of social comparison. Empirical articles were included if they met the following criteria: (1) available in English, (2) available on or before June 30, 2019, (3) ambulatory assessment of naturally occurring social comparison via self-report (i.e., participants use paper and pencil or technological devices to record social comparisons and associated experiences during daily life), (4) at least three assessments of social comparison per participant, and (5) assessments scheduled no more than 24 h apart OR instructions to record each time a comparison occurred.

**Figure 1:**
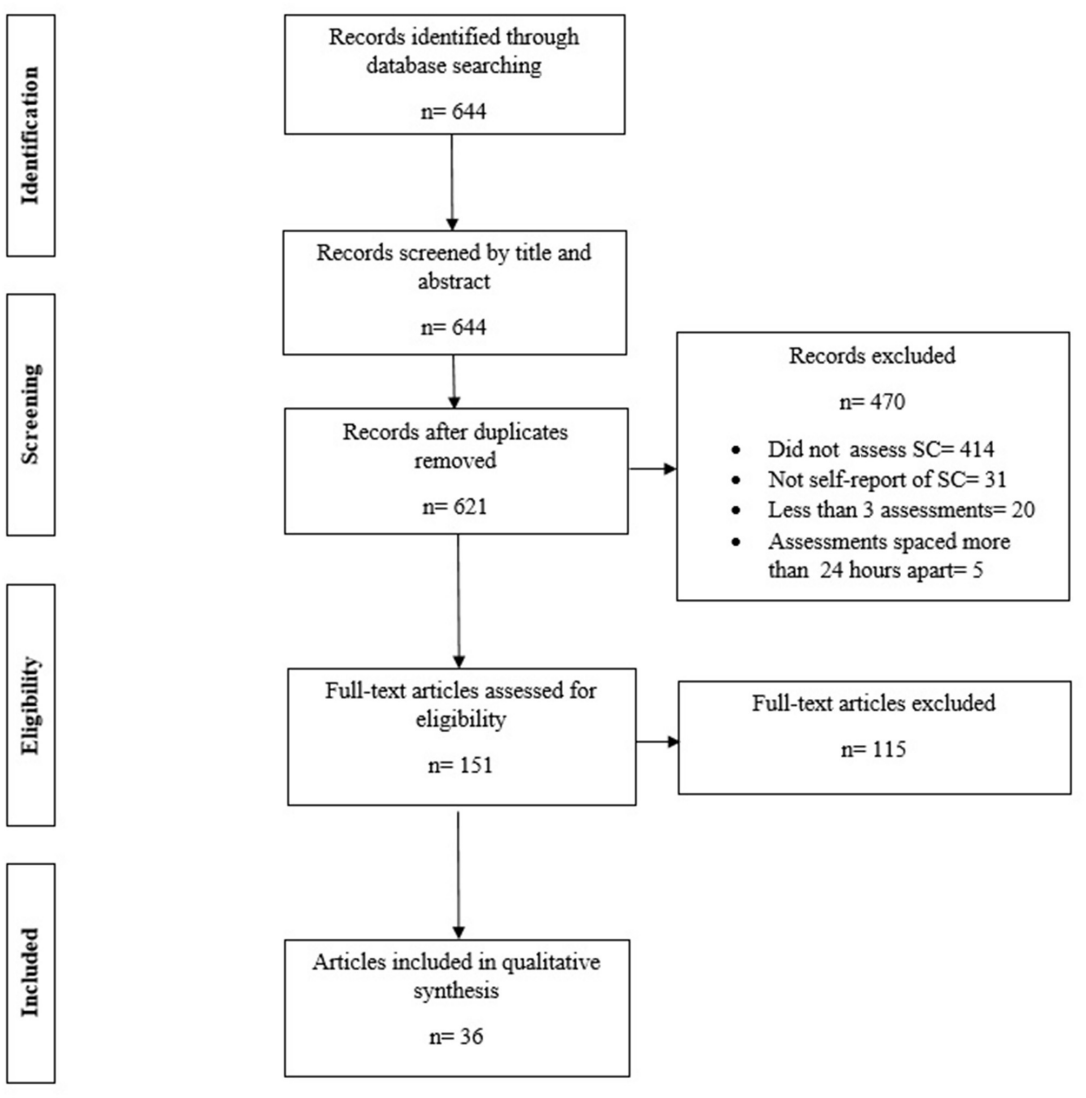
PRISMA-ScR flowchart; SC, social comparison.

The authors searched PubMed, PsycInfo, and CINAHL for relevant publications. Search terms were combinations of “social comparison” and “daily diary,” “diary,” “ecological assessment,” “intensive,” “repeated measures,” “event contingent,” or “experience sampling.” Resulting titles and abstracts were evaluated with respect to inclusion criteria; database and follow-up hand searches returned 644 individual articles. After removing 23 duplicates (resulting in 621 potential articles), four of the authors (MB, KP, LS, LT) reviewed the remaining titles and abstracts to determine inclusion. These authors were trained to recognize inclusion/exclusion criteria but were unaware of the review's specific research questions at the time of coding. After abstract review, 470 articles were excluded, leaving 151 articles for full text review. The same four authors (MB, KP, LS, LT) examined the full text of these articles to determine inclusion. A further 115 articles were excluded, leaving 36 in the final set of articles included for formal review and data extraction. Figure 1 shows our PRISMA-ScR flowchart describing the disposition of articles evaluated for inclusion, with a final total of 36. Multiple independent articles described findings from the same datasets in three cases (Fitzsimmons-Craft et al., [4 articles]; Leahey et al. [3 articles]; Thogersen-Ntourmani et al. [2 articles]) and two articles described more than one individually eligible dataset (Locke, 2003, Studies 1–3; Locke, 2007, Studies 1–2). To ensure that each unique set of methods (sample, recording method) counted once, we collapsed multiple papers from the same dataset (9 became 3) and added individual studies from multi-study publications (2 became 5). This resulted in 33 unique studies.

### Data Extraction

The final set of 33 included studies (36 published articles) were coded for the following characteristics: author(s), year of publication, the sample enrolled in the study (e.g., college students, older adults), whether assessment of social comparison as a predictor or outcome was the primary purpose of the study, the study context (e.g., body image, work performance, not specific), assessment design (i.e., daily diary vs. EMA), recording method (i.e., interval- vs. signal- vs. event-contingent assessment, or a combination), the recording modality (e.g., paper and pencil, smartphone), the number of assessments per day that included social comparison items, the aspects of comparison assessed (e.g., dimension, direction, identification), predictors of comparison occurrence or type (e.g., pre-comparison affect), the average number of comparisons recorded per assessment, and outcome variables assessed (e.g., affect, behavior). Four of the authors (MB, KP, LS, LT) each coded 6–7 articles for this information. Authors DA and JAM checked each set for accuracy, and discrepancies (which were minimal) were resolved by consensus. Due to the overlap of the sets of articles noted above, 33 was used as the denominator for all descriptive calculations.

## Results

The earliest study identified that used intensive, naturalistic assessment of social comparison was published in the early 1990s (Wheeler and Miyake, 1992). No other studies identified in our search were published until 2000 (Affleck et al., 2000), with the majority of studies appearing in published form between 2007 and 2017 (25). The most recent studies identified were published in early 2019 (Arigo et al., 2019b; Fuller-Tyszkiewicz et al., 2019).

### Research Question 1: Study Contexts and Populations of Interest

Social comparison was of primary interest in the vast majority of studies reviewed (*k* = 31; 94%). In the remaining two cases, social comparison was of secondary interest—as an influence on organizational citizenship behaviors (i.e., work activities focused on helping others; Spence et al., 2011) and “fat talk” (i.e., negative comments about weight; Mills and Fuller-Tyszkiewicz, 2018). Three studies enrolled wide subsets of the general population, including full-time employees (Spence et al., 2011), romantic partners (Pinkus et al., 2008), and adolescents (Lennarz et al., 2017; see Table 2). Specific populations of interest were women with fibromyalgia (Affleck et al., 2000) and ethnic minority students (non-Caucasian; Leach and Smith, 2006). The majority of studies enrolled college students, however (*k* = 19; 58%). Several of these studies enrolled only women (7) and assessed only appearance-related comparisons (5). Seven additional studies focused on appearance comparisons enrolled older women, with a total of 12 studies assessing appearance comparisons among women (36% of studies reviewed). Only one study focused on appearance comparisons enrolled both men and women (Pila et al., 2016), and no studies enrolled only men for this purpose. Further, one study of appearance comparison asked only about those toward upward targets (Pila et al., 2016). Across populations, one study focused exclusively on instances of being the target of someone else's upward comparisons (Koch and Metcalfe, 2011, Study 1), one assessed only downward comparisons (Affleck et al., 2000), and one assessed only experiences of negative-outcome comparisons (i.e., those that resulted in negative affect or self-views; Kashdan et al., 2014).

**Table 2:** Descriptive information for each article included in the present review (*k* = 36).

**Author (Year)**	**Sample**	**Study context**	**Recording method**	**Recall period**	**Number of reports per day**	**Number of assessment days**	**Recording modality**
Affleck et al. (2000)	89 women with fibromyalgia	Chronic pain/pain intensity	Interval	Current day	1	30	Paper and PDAs
Arigo et al. (2019b)	80 college women	Not specific	Interval	Current day	1	7	Any device that had internet access
Bogart et al. (2004)	98 college students	Not specific	Event	Most recent	N/A	3	Paper
Drutschinin et al. (2018)	161 women	Appearance	Signal	Since last prompt	6	7	iPhone
Fardouly et al. (2017)	146 college women	Appearance comparisons	Signal	Since last prompt	5	5	Any device that had internet access
Fitzsimmons-Craft (2017)	232 college women	Appearance-related comparisons; body, eating and exercise comparisons	Signal	Since last prompt	3	14	Personal electronic devices
Fitzsimmons-Craft et al. (2015)	232 college women	Body, eating, and exercise related social comparison	Signal	Since last prompt	3	14	Personal electronic devices
Fitzsimmons-Craft et al. (2016a)	232 college women	Appearance related; body, eating, and exercise social comparisons	Signal	Since last prompt	3	14	Personal electronic devices
Fitzsimmons-Craft et al. (2016b)	232 college women	Appearance related; body, eating, and exercise social comparisons	Signal	Since last prompt	3	14	Personal electronic devices
Fuller-Tyszkiewicz et al. (2019)	84 women aged 18-40	Appearance comparisons	Signal	Since last prompt	10	7	Phone
Kashdan et al. (2014)	172 college students	Daily negative social comparisons	Interval	Current day	1	21	Not specified
Koch and Metcalfe (2011), Study 1	49 participants	Upward social comparison	Event	Right now	N/A	14	Website (computer) & blank-pocket sized notebooks
Leach and Smith (2006)	32 ethnic minority students	“Ethnic minority students' comparisons to other ethnic minorities or to members of a high-status ethnic majority”	Signal	Most recent	3	7	Booklet (paper)
Leahey and Crowther (2008)	105 women	Appearance comparisons	Signal	Since last prompt	6	5	PDA
Leahey et al. (2011a)	160 women	Appearance comparisons	Signal	Since last prompt	6	5	Paper and pencil
Leahey et al. (2007)	153 women	Body-focused comparisons	Signal	Since last prompt	4	7	Not specified
Lennarz et al. (2017)	68 adolescents	Not specific	Signal	Right now	4 Friday & 9 on Saturday and Sunday	6	Phone
Locke and Nekich (2000)	157 college students	All	Event	Right now	N/A	7	Paper
Locke (2003), Study 1	106 college students	All	Event	Right now	N/A	N/A	Paper
Locke (2003), Study 2	109 college students	All	Event	Right now	N/A	N/A	Paper
Locke (2003), Study 3	191 college students	Not specific	Event	Right now	N/A	7	Paper
Locke (2005)	229 college students	Not specific	Event	Right now	N/A	N/A	Paper
Locke (2007), Study 1	130 college students	Not specific	Event	Right now,	N/A	7	Paper
Locke (2007), Study 2	132 college students	Not specific	Event	Right now	N/A	N/A	Paper
Mills and Fuller-Tyszkiewicz (2018)	135 women aged 18–40	Appearance comparisons	Signal	Since last prompt	6	7	Phone app
Myers et al. (2012)	91 college women	Appearance comparisons	Signal	Since last prompt	5	5	PDA
Patrick et al. (2004), Study 2	88 college women	Not specific	Event	Right now	N/A	10	Paper
Pila et al. (2016)	87 adults	Upward social comparisons (any and body-related)	Interval	Current day	N/A	7	Online survey
Pinkus et al. (2008), Study 1	95 couples 190 individuals)	Not specific	Signal	Since last prompt	6	14	PDA
Rancourt et al. (2015)	46 college women	Weight-related comparison	Signal	Since last prompt	6	5	PDA
Ridolfi et al. (2011)	93 college women	Appearance comparisons	Signal	Since last prompt	5	5	PDA
Rogers et al. (2017)	161 women	Appearance comparisons	Signal	Since last prompt	6	7	Phone app
Spence et al. (2011)	99 men and women	Coworker comparisons at work	Interval	Current day	1	14	Email
Steers et al. (2014), Study 2	154 college students	Not specific	Interval	Current day	1	14	Online if had access, others used paper
Summerville and Roese (2008)	34 adults	Not specific	Signal	Right now	7	14	PDA
Thøgersen-Ntoumani et al. (2017)	126 women	Appearance	Signal	Since waking up/last prompt	3	4	Phone
Thøgersen-Ntoumani et al. (2018)	126 women	Appearance	Signal	Since last report	3	7	Phone
Wheeler and Miyake (1992)	94 college students	Not specific	Event	Right now	N/A	14	Paper
Zuckerman and O'Loughlin (2006)	176 college students	Not specific	Interval	Current day	1	14	Online

*PDA, personal digital assistant (palmtop computer)*.

### Research Question 2: Methodological Factors

#### Recording Structures Across Studies

**Figure 2:**
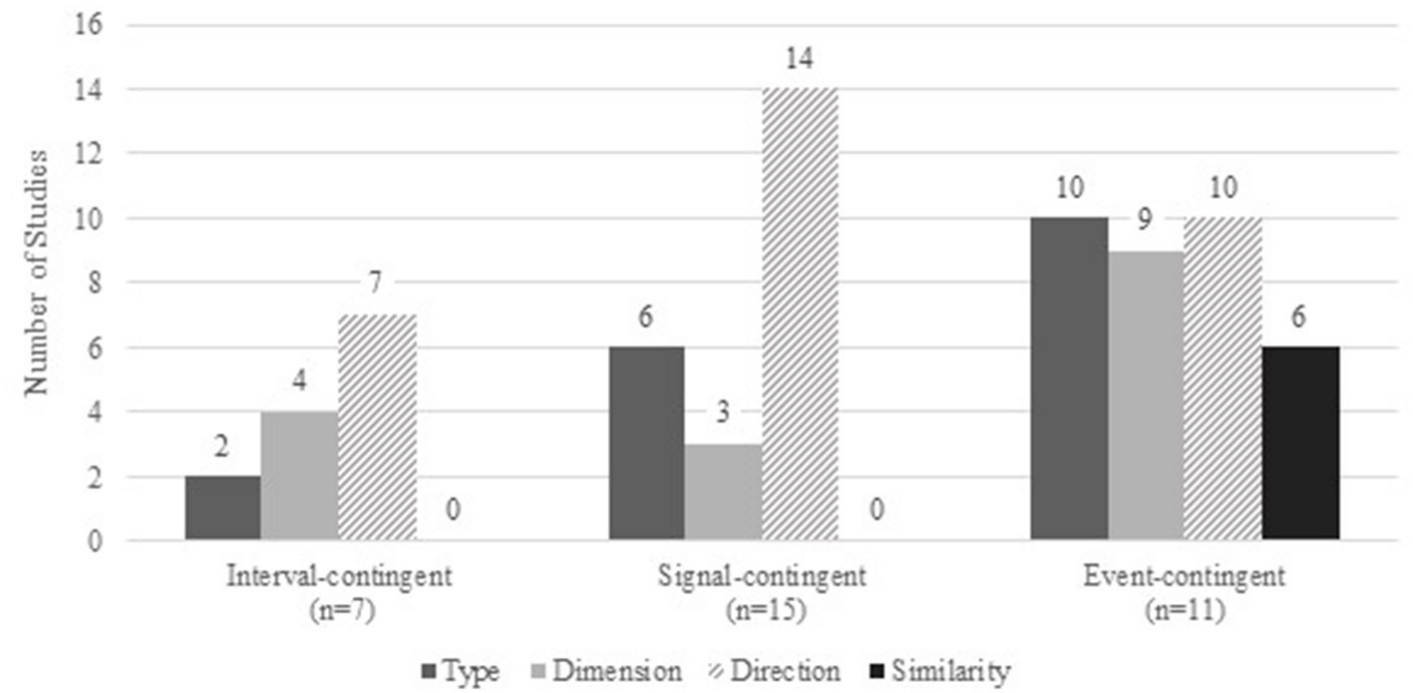
Summary of social comparison features assessed by study method.

The most frequently used method for collecting data on naturally occurring social comparisons was through signal-contingent recording (i.e., prompting participants to record recent comparisons, with multiple prompts within a day; *k* = 15; 46%), followed by event-contingent recording (i.e., participants recording each time they recognize that they have made a comparison; *k* = 11; 33%). The remaining studies used interval-contingent recording (i.e., recording after a set amount of time), at the end of the day (*k* = 7; 21%; see Table 2 and Figure 2). There was wide variety in the number of times per day participants were asked to respond in the signal-contingent protocols ranging from 3 times per day (4 studies) to 10 times per day (1 study). The most common frequency was 6 times per day (5 studies). One study varied the number based on day of the week, using 9 prompts on weekends and 4 prompts on weekdays (Lennarz et al., 2017). The number of recording days for signal-contingent studies ranged from 4 (1 study) to 14 (3 studies), with a mode of 7 (7 studies). The total number of assessments per person per signal-contingent study ranged from 21 to 98. Interval-contingent studies tended to ask participants to record their experiences once per day for longer durations. The number of assessment days per person was 7 (3 studies) to 30 days (1 study), with 14 days as the most common duration (4 studies). Event-contingent recording designs were more variable between studies, in that some studies specified a set number of days for each participant (e.g., 7 days; 3 studies) whereas others used the number of reported events to conclude the data collection (e.g., 10 events of comparison; Locke, 2005). In the latter cases, the duration of the study varied across participants.

#### Recording Method

All of the studies that used event-contingent recording were conducted using paper-based surveys, whereas studies using signal-contingent or interval-contingent recording were conducted via smartphones, personal computers (laptop/desktop), or palmtop computers (personal digital assistants, or PDAs). Three interval-contingent studies used a combination of paper and electronic reporting. Signal-contingent designs tended to use palmtop computers (12), though one study used paper reports (Leach and Smith, 2006) and another used online surveys (Fardouly et al., 2017), allowing participants to record from any internet-connected device. Consistent with recommendations for signal-based assessments (Smyth and Stone, 2003), several studies used “since the last” signal as the reporting time frame (12), whereas interval-contingent assessments used the “current day.” For studies employing event-contingent responding, the reporting interval was “right now.”

#### Recording Instructions, Item Wording, and Response Scales

Instructions and items used to assess social comparison varied across the methods of reporting. Studies using event-contingent methods were fairly consistent in response options, as the majority (7 out of 11) used the original or a modified version of the Rochester Social Comparison Record (RSCR; Wheeler and Miyake, 1992). This measure is completed each time a participant notices that they've made a comparison, and records are counted to determine the number of comparisons over a given time period. In addition to the date of the event, the RSCR asks participants to report on various features of each comparison (e.g., type of target, direction; described below). In contrast, event-contingent studies differed in their specific instructions to participants, with respect to the definition of a recordable comparison. Wheeler and Miyake (1992) and Patrick et al. (2004) stipulated that participants should only record instances of comparison to which they have a noticeable “psychological reaction.” All other researchers who used event-contingent methods used a broader definition, such that participants should record any instance of “similarities and/or differences between yourself and another person” (e.g., Koch and Metcalfe, 2011, Study 1; Locke, 2003, Studies 1-3; Locke and Nekich, 2000). In only one study were participants encouraged to consider comparisons with “imaginary” others, as well as those to real individuals (Patrick et al., 2004).

A small subset of studies using interval- or signal-contingent recording (*k* = 8; 24%) indicated that they provided participants with specific guidance in how to identify or define a comparison; these studies did not indicate that only comparisons with accompanying psychological reactions would count. All of these studies included items assessing the occurrence and/or frequency of comparisons, as well as a range of follow-up questions. One study using interval-contingent methods asked participants to estimate the total number of comparisons they made that day and record an integer of their estimate (Pila et al., 2016); 4 interval-contingent studies used rating scales to capture frequency [1–10 (Spence et al., 2011; Kashdan et al., 2014), 1–9 (Steers et al., 2014, Study 2), and 1–7 (Zuckerman and O'Loughlin, 2006)], and 1 study asked participants to indicate whether they had made any comparisons that day (yes/no; Arigo et al., 2019b). Studies using signal-contingent methods tended to begin each prompt by asking whether participants had made a social comparison (yes/no; 15) before asking follow-up questions about specific comparisons, with certain studies limiting the question to certain types of comparisons (e.g., appearance comparisons).

Across methods, the majority of included studies (*k* = 25; 76%) asked a number of follow-up questions regarding the “most recent” comparison to assess a range of features (described below). In 4 cases, study procedures described such follow-up questions but did not specify which comparison was assessed (Leahey et al., 2007; Myers et al., 2012; Mills and Fuller-Tyszkiewicz, 2018; Thøgersen-Ntoumani et al., 2018). A subset of signal-contingent studies used rating scales to capture the intensity or frequency with which participants had made comparisons (e.g., “Please slide the bar to indicate the level of [body] comparison behavior you have engaged in since the last time you were signaled, where 0 = No [body] comparisons and 100 = Constantly making [body] comparisons;” Fitzsimmons-Craft et al., 2015, 2016a,b) and did not appear to request additional information about a particular comparison.

### Research Question 3: Comparison Features

As expected, comparison target type, dimension, and direction were assessed in large subsets of studies reviewed (see Table 3 and Figure 2). Thirteen studies (39%) asked participants to report their relationship to the target (friend, family member, celebrity) to describe the target type. Two studies (6%) assessed only comparisons to a certain type of target: romantic partners (Pinkus et al., 2008, Study 1) and “the average college student of the same age and gender” as the participant (Zuckerman and O'Loughlin, 2006). Of note, only two studies (6%) asked participants to report the gender of their target (Koch and Metcalfe, 2011, Study 1; Wheeler and Miyake, 1992).

**Table 3:** Main features assessed in each article included in the present review (*k* = 36).

**Author (Year)**	**Features**
Affleck et al. (2000)	Downward comparisons of pain intensity only
Arigo et al. (2019b)	Target type
	Dimension
	Direction
Bogart et al. (2004)	Target type
	Dimension
	Direction
	Mode
Drutschinin et al. (2018)	Appearance comparisons only
	Direction
Fardouly et al. (2017)	Appearance comparisons only
	Direction
	Mode
Fitzsimmons-Craft (2017)	Target type
	Dimension
	Direction
Fitzsimmons-Craft et al. (2015)	Dimension (separate items for body, exercise, and eating)
Fitzsimmons-Craft et al. (2016a)	Dimension (separate items for body, exercise, and eating)
Fitzsimmons-Craft et al. (2016b)	Dimension (separate items for body, exercise, and eating)
Fuller-Tyszkiewicz et al. (2019)	Body comparisons only
	Direction
Kashdan et al. (2014)	Direction
Koch and Metcalfe (2011), Study 1	Self-used as upward target only
	Target type
	Target gender
	Dimension
Leach and Smith (2006)	Dimension
Leahey and Crowther (2008)	Body shape/weight comparisons only
	Target type
	Direction
Leahey et al. (2011a)	Body shape/weight comparisons only
	Direction
Leahey et al. (2007)	Body shape/weight comparisons only
	Direction
Lennarz et al. (2017)	Direction
Locke and Nekich (2000)	Target type
	Dimension
	Mode (1)
	Direction
	Similarity
Locke (2003), Study 1	Target type
	Dimension
	Direction
	Similarity
Locke (2003), Study 2	Target type
	Dimension
	Direction
	Mode
	Similarity
Locke (2003), Study 3	Target type
	Direction
	Mode
	Similarity
Locke (2005)	Target type
	Direction
	Similarity
Locke (2007), Study 1	Target type
	Direction
	Mode (1)
Locke (2007), Study 2	Target type
	Direction
	Similarity
Mills and Fuller-Tyszkiewicz (2018)	Appearance comparisons only
	Direction
Myers et al. (2012)	Appearance comparisons only
	Direction
Patrick et al. (2004), Study 2	Target type
	Dimension
	Direction
	Mode
Pila et al. (2016)	Upward comparisons only
	Dimension (body vs. other)
Pinkus et al. (2008), Study 1	Comparisons to significant other only
	Target gender
	Dimension
	Direction
Rancourt et al. (2015)	Body weight/shape comparisons only
	Target type
	Direction
Ridolfi et al. (2011)	Body weight/shape comparisons only
	Target type
	Direction
Rogers et al. (2017)	Body comparisons only
	Direction
Spence et al. (2011)	Dimension (work-related only)
	Direction
Steers et al. (2014), Study 2	Dimension
	Direction
Summerville and Roese (2008)	Direction
Thøgersen-Ntoumani et al. (2017)	Appearance comparisons only
	Direction
Thøgersen-Ntoumani et al. (2018)	Appearance comparisons only
	Direction
Wheeler and Miyake (1992)	Target type
	Target gender
	Dimension
	Direction
	Mode
Zuckerman and O'Loughlin (2006)	Comparison to the average college student of same age and gender only
	Dimension
	Direction

Dimension was assessed in 17 studies (52%), using checklists or open-ended responses. Checklist options most often included academic performance, appearance, wealth, personality, abilities, and opinions. Of the remaining 16 studies, 13 asked about appearance comparisons exclusively. These were specified as “appearance comparisons” (broad), “body comparisons,” and “weight/shape comparisons;” only one study allowed participants to select the specific appearance dimension (e.g., weight, shape, muscularity/level of tone, physical abilities; Fitzsimmons-Craft, 2017). Two studies asked specifically about comparisons of health behaviors, which might also relate to appearance. The first offered a category called “health habits (e.g., physical activity, eating behavior)” (Arigo et al., 2019b), and the second asked about eating and exercise comparisons with unique items (described in Fitzsimmons-Craft et al., 2015). Although the RSCR does not offer “eating” as a comparison category, Wheeler and Miyake's (1992) initial study using this measure indicated their ability to assess eating comparisons. It is likely that these were coded from free responses to an “other” category, although the procedure does not explicitly state this.

The most prevalent comparison feature was direction, however. This feature was assessed in 30 studies (91%); the majority of these studies (23) allowed researchers to capture upward, downward, and lateral comparisons, whereas a smaller subset assessed only upward and downward comparisons (7). Importantly, response options for direction were most often offered on a continuous scale (e.g., *I am much worse than the target* to *I am much better than the target*; 15 studies), with the scale midpoint representing lateral comparisons. In some cases, these continuous responses were recoded to form upward, downward, and lateral categories, and these were used in statistical analyses (e.g., Leahey and Crowther, 2008). As noted, additional studies assessed only upward comparisons (Pila et al., 2016) or only downward comparisons (Affleck et al., 2000).

“Similarity” was assessed in a small subset of studies (*k* = 6; 18%), typically framed as whether the participant perceived similarity or dissimilarity between the self and the target (e.g., similar, dissimilar, or neither; Locke and Nekich, 2000). Of note, this language also was used to frame perceptions of direction, with the response options specifying the intent of the item (i.e., similarity with respect to how much better or worse off the respondent is than the target; Wheeler and Miyake, 1992). Similarly, a subset (8 studies) assessed the mode or setting of comparison (e.g., face-to-face interaction, exposure via media), with 5 studies explicitly assessing comparisons via social media platforms (Patrick et al., 2004; Leahey and Crowther, 2008; Fardouly et al., 2017, Study 2; Ridolfi et al., 2011; Steers et al., 2014; Rancourt et al., 2015, Study 2). Other comparison features assessed included target's ethnicity (Leach and Smith, 2006), the participant's location and the “density” of people present at the time of comparison (Fitzsimmons-Craft, 2017), the importance or desirability of the comparison dimension to the participant (Koch and Metcalfe, 2011, Study 1; Locke, 2003, Study 1), how helpful the participant perceived the comparison to be (Locke, 2003, Study 2), whether the participant had compared to the target in the past or expected to in the future (Locke, 2007, Study 1), the participant's main concern during the comparison (Locke, 2007, Study 2; Locke and Nekich, 2000), the participant's reason for making the comparison (Patrick et al., 2004), and whether the comparison was made deliberately or automatically (Locke, 2005).

### Research Question 4: Comparison Frequency

A goal of this review was to describe the frequency (or range of frequencies) with which participants in intensive assessment studies reported making comparisons. As noted, however, studies varied in their approach to assessing comparison frequency; some asked only whether a comparison had occurred over a given time frame (yes/no), whereas others requested an estimate of the number of comparisons made in a specific time frame. Four studies reported the average number of comparisons per recording period (with corresponding standard errors/deviations; 12%) and two (6%) reported the overall average per person (with corresponding standard errors/deviations). An additional 12 studies (45%) appeared to provide enough information to estimate an average number of comparisons recorded per day, although the variability in day-to-day reporting would be more difficult to estimate. For example, 4 studies indicated the average number of days it took participants to reach a pre-identified number of assessments, in some event-contingent studies (e.g., Locke, 2003, Study 1), and a subset of these provided averages for subgroups only, rather than the full sample (e.g., Leahey et al., 2007).

In most cases, however, it was not clear whether averages or variability estimates (e.g., standard deviations) were between- or within-person, which reflect distinct aspects of comparison. These represent the amount of variability between participants (i.e., stable throughout assessments) vs. within participants (changing within the same participant over time), and thus, could provide important insight into questions about within-person change (e.g., in affective response to comparison). Only a small number of studies explicitly described testing for comparison variability at the between vs. within-person levels (e.g., intraclass correlation coefficients; Locke and Nekich, 2000; Spence et al., 2011; Steers et al., 2014; Pila et al., 2016, Study 2). These studies documented within-person variability in comparison frequency, ranging from 50 to 95% of total variability (We note that these estimates also include error variance.). As their assessments used different time scales, however, it is not possible to draw strong conclusions about variability in frequency across studies (see Podsakoff et al., 2019). Finally, a subset of studies reported on the absolute or relative frequencies of recorded comparisons with specific features, such as the most common target types (e.g., Wheeler and Miyake, 1992; Patrick et al., 2004; Fardouly et al., 2017), dimension(s) (e.g., Wheeler and Miyake, 1992; Patrick et al., 2004; Fitzsimmons-Craft, 2017) or direction (e.g., Locke, 2003, Study 3; Locke and Nekich, 2000; Pinkus et al., 2008, Study 1; Wheeler and Miyake, 1992; Spence et al., 2011).

### Research Question 5: Predictors and Outcomes of Comparison

#### Predictors of Comparison Occurrence or Frequency

Seventeen studies (32%) evaluated between or within-person predictors of comparison reports (occurrence, frequency, or type). Between-person predictors included gender (9 studies), self-esteem (3 studies), body dissatisfaction (2 studies), age (2 studies). Tendency toward jealousy, body mass index, narcissistic personality traits, feminist beliefs, and agency were included as predictors in one study each. As noted, however, our primary interest for this research question was in within-person relations, which describe when (or under what circumstances) comparisons are most likely to occur, and cannot be inferred from between-person relations. A subset of studies described findings related to within-person predictors of comparison occurrence, though these predictors were idiosyncratic. At the day level, these included time spent on Facebook (Steers et al., 2014, Study 2), engaging in sexual activity with one's partner and feelings of connectedness (Kashdan et al., 2014), and pain intensity and positive and negative affect (Affleck et al., 2000). At the moment or event levels, predictors of interest were positive and negative affect (Wheeler and Miyake, 1992; Thøgersen-Ntoumani et al., 2018), comparison setting (i.e., during social interactions vs. alone—Locke, 2003, Study 3; Locke and Nekich, 2000; who was present—Lennarz et al., 2017), and state body dissatisfaction (Rogers et al., 2017).

#### Consequences of Making Social Comparisons

Similarly, our aim was to describe the within-person consequences of comparison that have been assessed in the natural environment (i.e., what happens when a person makes a comparison, or makes a certain type of comparison?). Within-person effects describe temporal relations between comparison outcomes that also cannot be inferred from between-person findings. The relevant outcomes assessed in the articles reviewed included self-reports of affect, internal experiences (e.g., thoughts, motivation), and behaviors, as well as objectively assessed behavioral engagement. Among studies focused on appearance comparisons, primary outcomes included body/appearance (dis)satisfaction (8 studies), reported engagement in disordered eating behaviors or physical activity (7 studies), thoughts about disordered eating behaviors (e.g., binge eating, restriction) or exercise (5 studies), affect (4 studies), guilt (3 studies), and social physique anxiety and drive for thinness (1 study). Outside of the context of appearance comparisons, 12 studies assessed post-comparison affect, two of which also captured pre-comparison affect and were able to control for this variable in subsequent tests (Wheeler and Miyake, 1992; Leach and Smith, 2006). Additional internal experiences of interest were self-esteem/confidence (5 studies), subjective well-being, depressive symptoms, jealousy, and feeling connected to others (1 study each). Two studies captured self-reports of engaging in specific behaviors: organizational citizenship behaviors (Spence et al., 2011) and physical activity/exercise (Pila et al., 2016). Only one study assessed behavior objectively, using a wristworn sensor to measure engagement in physical activity (Arigo et al., 2019b).

## Discussion

Many decades of research and theory on social comparison have revealed considerable nuance and complexity in this process, particularly in natural settings and over brief periods of time. Methods that provide intensive, within-person assessments in the natural environment may capture data that help clarify some of these important issues. This would be informative for both a basic understanding of human social and cognitive experiences and for designing tailored environments or interventions to promote positive outcomes. Yet, the extent to which this type of assessment is useful depends on the specific research design; a single study can assess only so many unique characteristics, predictors, and outcomes of comparisons (without placing undue burden on participants), under the constraints of the selected signal or recording timeframe. Decisions made about the design could shape a study's outcome(s), and should be considered carefully in the context of the specific research questions at hand. Here, we summarize findings from our review of extant studies and offer recommendations for key points to consider in planning future research with intensive assessment of social comparisons in the natural environment. We summarize our recommendations in Table 4.

**Table 4:** Summary of recommendations for future research using intensive assessment methods to study social comparison.

**Category**	**Considerations**	**Recommendations**	**Where is additional work needed**
Conceptual definition of social comparison	How will social comparison be defined? - Will comparisons “count” if they are not subjectively associated with a psychological response?	Defining social comparison more broadly (vs. associated with psychological responses); however, this depends on the research question	To determine the extent to which different definitions of comparison lead to different reporting patterns
	How will participants be taught to recognize comparisons in their daily lives?	Interactive instruction in how to recognize comparisons may reduce heterogeneity in identification and reporting; normalizing comparison may reduce hesitation to report	To determine whether giving instructions in a group setting affects reporting
	Will instructions be given individually or in a group?	- The instruction process should be described in detail in published reports	
Sample characteristics	What is the rationale for studying social comparison in a given population, and how narrowly should the population be defined?	Rationale should be clear from the outset and should be described in published reports	To understand comparisons other than those based on appearance among young women and all types of social comparison in more diverse samples
	What type(s) of comparison will be assessed and why?		Specifically, to understand social comparison (across dimensions) in the following groups: - Adults over the age of 25 - Men and trans/non-binary individuals (particularly regarding appearance comparison) - Individuals with chronic illness/health conditions - Those interested in behavior change (to elucidate how comparisons function in the behavior change process)
Recording and data collection parameters	What type of recording method will be used (signal-, interval-, or event-contingent)? What is the recording modality (paper, smartphone app, web link)?	Base these on:-What is known and/or proposed about the likely frequency of the type(s) of comparison of interest (evidence and theory) - Maximizing reach, ease, and accuracy while minimizing participant burden - Pilot work with the population of interest	To determine whether different types and frequencies of recording lead to differing response patterns
	How many total days of recording? Are the days consecutive or does the period include breaks? How many times per day will participant record (signal- and interval-contingent)? Will the number of times per day be consistent across days, or will it change?	Specific to the population of interest, select the recording frequency that would maximize accuracy and power for planned analyses while minimizing aggregation/recall bias *and* participant burden; rationale should be described in published reports- If possible, build in assessment of reactivity	To determine the extent of reactivity to recording social comparisons and related experiences (e.g., consequent affect)
Features assessed	Which features are critical to answering the research question?	Assess target gender and relation to participant	To investigate the influence of: - Mode (particularly social media) - Reason for making a comparison or selecting a particular target - Perceived utility of a comparison - Real vs. imaginary targets - Deliberate vs. automatic comparison - Identification/contrast processes
	Which features are likely to moderate or place boundaries on the primary effects in question?	Assess perceived direction and degree of similarity separately	
		Assess identification and contrast directly (rather than inferring from affective response)—additional work is needed here	
		Unless the research question is specific to a particular dimension, allow for a wide range and assess with high granularity (e.g., “appearance” could mean weight, shape, overall fitness/physique, facial attractiveness, etc.)	
Predictors and outcomes of social comparison	Is the research question(s) about the comparison process or the effect of comparisons on another variable (or both)?	Base this on a broad understanding of social comparison processes, rather than knowledge of comparison in a single domain	To examine: - Within-person variability in the frequency of comparison - Temporal patterns of comparison occurrence - Effect of comparisons on objectively assessed behavior
		Most commonly assessed predictors are between-person (e.g., self-esteem, body satisfaction, gender)	
		Most commonly assessed outcomes are affective response, body satisfaction, thoughts about or reports of eating/dieting/exercising (within-person)	
		Report on variability at the between- and within-person levels and specify which is being reported	

### Whom Are We Studying, and for What Reasons?

Intensive assessment has been applied in both broad and narrow samples. The rationales for focusing on specific groups such as adolescents, adults with full-time employment, and women with fibromyalgia naturally related to the specific outcomes of interest, such as jealousy, work behaviors, and pain experiences, respectively. Indeed, published work demonstrates that social comparisons are common in these populations and may be associated with key health, well-being, and performance outcomes. As each of these populations and outcomes have been studied using intensive assessment only once, replication and extension of the reported findings would be informative. As is common in psychological science, however, the majority of existing studies focused on convenience samples of college students. Given that young adults tend to report stronger tendencies toward comparison than older adults (Callan et al., 2015), future attempts to draw conclusions about the likelihood, frequency, or consequences of comparisons in non-college samples may be skewed by this overrepresentation.

Similarly, large subsets of existing work on intensive assessment have focused only on comparisons of appearance, and only one study of appearance comparisons has enrolled men to study this process. Although these also are limitations of the appearance comparison literature more broadly, their presence in intensive assessment work presents unique challenges. For example, the overall social comparison literature suggests a discrepancy between the effects of appearance comparisons and comparisons in other domains. Upward appearance comparisons almost universally lead to negative outcomes (e.g., increased negative affect or body dissatisfaction), while downward appearance comparisons do not seem to have a “symmetrical” positive effect (Lin and Kulik, 2002). In contrast, with respect to many non-appearance dimensions (e.g., chronic illness prognosis, work performance, positive and negative affect more broadly), both upward and downward comparisons show positive *and* negative effects, of varying intensities (Buunk et al., 1990; Van der Zee et al., 2000; Arigo et al., 2015). The broader literature has not yet been able to determine the features or contexts of comparison that determine positive vs. negative affect, and focusing on within-person processes using intensive assessment could be useful toward this end. Yet, overemphasis on naturally occurring appearance comparisons using these methods, rather than on other types of comparisons, may skew conclusions toward appearance-related patterns (e.g., upward comparisons lead to negative affect). This could mask broader, and important, within-person variability in affective response and other outcomes of interest.

Further, a focus on women in the appearance domain reflects the historical view that body dissatisfaction and disordered eating behaviors are more common among (or exclusive to) women. Although these experiences remain slightly more common among women than men, recent work has demonstrated that they increasingly occur among men (Turel et al., 2018) and trans/non-binary individuals (Sequeira et al., 2018). Little is known about how men and trans/non- binary individuals make and respond to comparisons—appearance-based or otherwise—in their natural environments, limiting the potential for understanding the range of comparison responses and for tailored intervention in these groups.

Additional populations that warrant increased attention using intensive assessment of social comparison include individuals with chronic illnesses and those attempting to change their behaviors. People with illnesses such as cancer, type 2 diabetes, and cardiovascular disease experience ongoing threats to their health that can increase the utility of social comparison, as comparisons can provide comfort, inspiration, and guidance for self-care (Kulik et al., 1993; Van der Zee et al., 1998a; Stanton et al., 1999). Research using between-person methods, such as randomized experiments, behavioral selection, and retrospective self-report, show between-person variability in comparison target preference and affective response (Arigo et al., 2014). The present review identified only one intensive assessment study of adults with a chronic illness (fibromyalgia; Affleck et al., 2000), and the sample was restricted to women. Thus, the extent of within-person variability in comparison among individuals with chronic illnesses is not yet clear, and this variability could provide insight into a critical component of health in these at-risk groups.

In addition, people interested in modifying their behaviors may use a range of others as role models and information sources, particularly if they join group programs or use digital support tools with social networking features (Direito et al., 2014). Social comparison has been identified as an important and potentially effective behavior change technique for a range of outcomes (Abraham and Michie, 2008; Olander et al., 2013). Different individuals may need different types of comparisons to motivate change (cf. Schokker et al., 2010), however, and people also may need different types of comparisons at different times (Arigo and Suls, 2018). Increased use of intensive assessment, both prior to and during focused behavior change efforts, would be useful for further understanding within-person variability in change processes and for optimizing social comparison features of intervention programs. As described further below, it would be extremely helpful for future work in this area to provide additional information about within-person variability in comparison frequency and outcomes.

### How Are We Designing Intensive Assessment Studies?

As evidenced by the number of logistical approaches described in this review, intensive assessment of social comparison has occurred using a range of data collection parameters. Specifics such as the number of assessment days, the frequency of assessments (i.e., how many per day, consecutive vs. nonconsecutive days, consistent number of assessments per day vs. changing), the recording modality (i.e., paper vs. technological device), and the recording or prompt method (i.e., event- vs. signal vs. interval-contingent) have varied widely across studies. In studies published to date, the most popular methods were signal-contingent recording using electronic services (e.g., PDA, smartphone, email), 6 signals per day, and 7 consecutive days of assessment. The rationale for the specific number of days or assessments was not always clear, however, and deserves more careful consideration, as the most common methods may not be appropriate for all research questions.

Conceptual decisions about what is considered a social comparison and what dimensions of social comparison are critical to the aims of the study should guide methodological decisions about timing of assessments, how recording will be carried out, and obtaining quality data from participants (cf. Stone and Shiffman, 2002). Researchers must identify the specific type of design that best fits their research question (i.e., interval-, signal-, or event-contingent) and then select the recording method that can limit participant burden and maximize reporting compliance. For researchers interested in assessing the frequency of the occurrence of social comparisons, it is critical to build this question effectively into protocols. All recording methods (i.e., interval-, signal-, or event-contingent) could capture this information, although the questions should be framed slightly differently for each method. For example, with event-contingent recording, the number of records completed is intended to capture the natural frequency of salient comparisons. With interval- and signal-contingent methods, researchers should use self-report items that align with their frequency-related research questions (e.g., number of comparisons in a time frame vs. occurrence or not).

The decision between interval- and signal-contingent recording will vary based on a given researcher's predictions how often the comparisons of primary interest are likely to occur. Researchers examining more frequently occurring comparisons may prefer the shorter retrospection periods of signal-contingent recording, whereas those examining less frequently occurring comparisons could leverage the reduced burden of interval-contingent recording. Similarly, researchers interested in the behavioral and emotional consequences of comparisons should consider briefer response windows, such as those in signal-contingent recording, to ensure timely assessment of consequences. Repeated assessments within a shorter window of time also provide opportunities to examine immediate (i.e., same report) consequences, as well as consequences later in the study window that could imply a delayed response to the comparison (e.g., lagged effects; Larson and Almeida, 1999; Schuurman et al., 2016).

#### Recording Modality

Previous work demonstrates the unreliability of paper-based reporting methods (Stone et al., 2002, 2003), and the present review indicates that paper-based recording of social comparison in the natural environment decreased since initial studies in the 1990s and early 2000s. Technologies such as PDAs and smartphones have the advantage of providing time stamps to verify when the record was completed (and may be more efficient for recording comparisons than paper), although some participants may be less inclined to type (vs. write) open-ended responses. Of available technologies, allowing participants to use their personal smartphones may seem optimal, as it limits the new resources necessary to conduct the study and generally is perceived as convenient (Kuntsche and Labhart, 2013). This method also offers a range of distribution options, such as by sending survey links via text message or email, or recording responses in a downloadable app, but has clear disadvantages. For example, these methods often require participants to use their own (potentially limited) data plans to access internet services and require additional attention to privacy and security. Further, smartphone ownership may not be prevalent in all populations of interest, and reliance on personal devices in these situations will result in meaningful selection bias if alternatives are not provided. Here, knowledge of the population and pilot work can inform decisions.

#### Reporting Heterogeneity

As noted, we encountered difficulty describing the frequency of comparisons due to discrepancies in results reporting, which was unexpected. Many published papers do not provide basic descriptive statistics (and/or do not clearly specify the level for descriptives that are reported) which preclude strong conclusions about frequency of naturalistic comparisons, day-to-day or moment-to-moment variability in comparison occurrence, or responses to specific types of comparisons. This information could be critical to mapping the comparison process accurately and to translating this information to applied contexts. The limited information we could glean from existing studies appears to support the presence of considerable within-person variability in comparison frequency. It is not yet possible to draw strong conclusions about this variability across studies, or to speak to within-person variability in related aspects of comparison (e.g., types of targets, affective response). In future work, we recommend that researchers consider the unit(s) of analysis (i.e., person, day, and/or moment) and provide descriptive information that matches the lowest unit of analysis.

As an example, consider a signal-contingent design with 4 assessments per day across 1 week, with a question about the frequency of social comparisons. Indicating that participants reported an average of 2 comparisons at each momentary assessment would provide researchers with an estimate of the average number of responses per person per day (~8), as well as the total number per person for the week of assessments (~56). Additionally, reporting that there was a standard deviation of 1 comparison at the momentary level further extends the information that can be extrapolated. We could then learn that, for this hypothetical study, most participants reported a range of 1–3 comparisons at each momentary assessment for a range of 4–12 comparisons per day and a range of 28–84 comparisons across the study duration of 1 week. Further, reporting such descriptives for identification/contrast and comparison outcomes, as well as for raw occurrence, would be useful information for researchers planning similar studies or clinicians attempting to identify the role of social comparisons in their intervention protocol. Providing descriptive details for individual types of comparisons and for specific subgroups of interest within the study design can further inform the literature on social comparisons.

#### Features of Interest

Consistent with the broader literature on social comparison, features of comparison often captured in intensive assessment of social comparison were target type, direction, and dimension. Less than half of the studies reviewed assessed target type. As existing evidence suggests that a person's relationship or perceived closeness to the target is associated with comparison response (and thereby, the utility of a comparison for achieving a particular purpose; Zell and Alicke, 2010), it is possible that this piece of potentially important information is missing from intensive assessment studies. In contrast, the popularity of assessing direction may reflect a widespread notion that direction is key to understanding the effect of comparisons on key outcomes. Although Festinger (1954) described a “unidirectional drive upward” in the group settings that were the focus of his original theory, subsequent work has demonstrated that (1) comparison is an intrapsychic process that does not require the presence of a group (Schachter, 1959), (2) the potential utility and disadvantages of both downward and lateral comparison (Wills, 1981; Mahler et al., 1995; Alicke, 2000), and (3) that direction may reflect not only a categorical perception but also one of scale (Wheeler et al., 1969; Wood, 1989). Existing work using intensive assessment has incorporated these insights to varying degrees, though the rationales for doing so (e.g., why using continuous vs. categorical responses for direction were most appropriate for the specific research question) were not entirely clear.

More recent work also has shown that people make comparisons on dimensions of the self beyond abilities and opinions, on which Festinger focused (Suls, 1986; Heidrich and Ryff, 1993; Arigo et al., 2014), and that individuals differ in their preferences and reactions to comparisons on distinct dimensions (Bennenbroek et al., 2002; Derlega et al., 2008). Further, a given domain of the self or behavior may actually encompass several specific dimensions, which do not all have the same value to a particular person. For example, exercise comparisons may be made on the total number of steps per day or exercise sessions per week, as well as on overall physical fitness or progress toward a goal (Harrison et al., 2015). Similarly, “appearance” comparisons may be made on overall level of body weight or shape, clothing size, general level of attractiveness, or muscularity; “eating” comparisons may be made on quantity, quality, or frequency of eating behavior; and “personality” comparisons may be made on a host of different traits or behavioral demonstrations of such traits. Although dimension commonly was captured in intensive assessment studies, very few provided participants or readers with these levels of specificity. This omission may reflect an effort to limit participant reporting burden, as increasing the number of options can amplify cognitive load (Yan and Tourangeau, 2008). In order to advance the current understanding of naturally occurring comparisons, however, it may be important to improve the granularity of response options with respect to dimension—even in studies that focus on a particular comparison domain (e.g., appearance).

Additional features of interest in existing intensive assessment studies were mode, similarity, and a range of characteristics unique to one or two studies (e.g., how helpful the participant perceived the comparison to be; Locke, 2003, Study 2). Capturing variability in mode reflects that people do not have to encounter targets face to face; targets can appear at a greater distance, such as over the telephone, and many of today's comparisons happen via social media. Since 2011, as the popularity of social media has increased, the frequency of explicit reference to social media in intensive assessment studies also has increased. Comparisons on these platforms have been shown to impact physical activity, self-esteem, and overall well-being (Dibb, 2019; Divine et al., 2019; Schmuck et al., 2019), although such comparisons may be missed (particularly by those who spend less time on social media) if not explicitly referenced in study training materials or assessment items. In some cases, people may not even have to encounter their comparison target in any tangible sense. There is some evidence that people create comparison targets to fit the characteristics that suit their goals (e.g., self-enhancement), suggesting that targets can be imaginary, and that comparisons to these targets are associated with some outcomes of interest (e.g., health outcomes; Wood et al., 1985). As only one intensive assessment study reviewed here explicitly indicated that targets could be imaginary (Patrick et al., 2004), such targets represent an additional category that may be missed without specific introduction or assessment. Intensive assessment of comparisons to imaginary targets also would provide insight into their frequency daily life.

Identification and contrast, often described as “perceived similarity” to the target, represent recent developments in social comparison theory (Buunk and Ybema, 1997). Although similarity was of interest in a subset of intensive assessment studies, we did not find evidence that it was used in a way that reflects identification and contrast processes as they were theorized to work (i.e., the comparer's emphasis on similarities and/or differences between the self and the target at the time of comparison). In studies that did assess “similarity,” this construct was operationalized in two distinct ways: to describe either the participant's overall perception of similarity (similar, dissimilar, or neither; Locke and Nekich, 2000) or to describe a directional scale (e.g., how much better or worse off the comparer perceives the target to be, which actually captures direction; Wheeler and Miyake, 1992). In some studies, these even were mixed together as multiple-choice options (e.g., Locke, 2003, Study 1), potentially creating additional confusion.

Empirical evidence indicates that identification and contrast are distinct aspects of a single comparison and that they may account for between-person variability in the effects of upward and downward comparisons (Van der Zee et al., 2000; Arigo et al., 2015). Findings from this review suggest that identification and contrast have not yet been included in intensive assessment studies, however. It is possible that identification and contrast represent a missing link that could help to explain why both upward and downward comparisons can have positive and negative affective consequences—people identify and contrast with specific targets to different extents at different times, leading to variability in their affective (and perhaps other) responses. As such, greater attention to this aspect of comparison in intensive assessment studies, using clear definitions and consistent terminology and/or measurement methods, may help to shed light on a critical but understudied aspect of comparison at the within-person level.

### Which Predictors and Outcomes Are We Including?

Despite the within-person emphasis of many intensive assessment studies, the majority of predictors of comparison occurrence, frequency, or type were those traditionally considered stable, between-person constructs (e.g., self-esteem, gender). Studies that did use within-person predictors focused on immediate affect (e.g., Wheeler and Miyake, 1992) and experiences specific to the context of the study (e.g., sexual activity; Kashdan et al., 2014). Of note, we did not find evidence of interest in more foundational descriptive questions, such as during which days of week or times of day comparisons were most likely to occur. The majority of existing intensive assessment studies focused on research questions about the outcomes of comparison, with a wider range of constructs assessed. Across research contexts and populations, however, there was a heavy emphasis on affect and other internal experiences (e.g., body satisfaction, thoughts, motivation) as comparison outcomes. Affect can be an indicator of how a person interprets a comparison (i.e., identification and contrast), and often has been the assessment method of choice for this construct (cf. Van der Zee et al., 2000). But immediate affect does not necessarily translate directly to overall well-being or behavior. For example, regularly making upward comparisons that provide momentary anxiety or discouragement—but also provide useful information about how to improve—could lend itself to achieving high life satisfaction, well-being, and goal-directed behavior over time (Wood, 1989; Collins, 1996). Thus, assessing affect as a proxy for other variables should be avoided; it would be preferable to assess the variable of interest directly, as efficiently as possible.

Many existing intensive assessment studies were conducted in the traditions of social psychology, which has typically emphasized behavioral outcomes less often than emotional or motivational outcomes (Baumeister et al., 2007). As such, assessment of behavioral outcomes appeared more often in studies designed for clinical or other applied contexts (e.g., disordered eating, workplace engagement) than in those focused primarily on understanding the comparison process (e.g., Locke, 2005). Across studies that did focus on behavioral outcomes, however, behavior was measured almost exclusively via self-report, and only one study used more objective assessment of behavior (physical activity; Arigo et al., 2019b). Reports of behavioral engagement are an improvement over assessing only motivation or thoughts about behaviors (as in several appearance comparison studies). Yet, given the known gaps between motivation or intentions and actual behaviors (Sheeran and Webb, 2016), and in light of new technologies that make at least some aspects of ambulatory behavioral assessment more affordable and less burdensome, relations between comparisons and objectively assessed behavioral outcomes represents a new and exciting frontier for intensive assessment research.

More broadly, if the primary within-person research question focuses on the consequences of comparison, it is critical that researchers consider the various features of comparison that may moderate its effects in the moment. For example, appearance comparisons may have negative consequences, but typically this is restricted to upward comparisons and is strongest when the target is a model (vs. a peer). In contrast, upward comparisons of athletic ability, particularly if the target is a peer, may have positive consequences. Unless the research question is restricted to (and researchers decide to assess only) a very specific type of comparison, understanding the “effect” of a comparison in daily life requires assessment of its occurrence, direction, dimension, and target type, among other features hypothesized to play a role. Greater attention to the boundary conditions of comparison effects in intensive assessment studies could help to map more specific temporal patterns at the (sub)group, individual, day, and moment levels.

To select the most appropriate comparison features for intensive assessment, researchers should have a general understanding of social comparison theory and evidence, not only in their population or domain of interest but more broadly. This will ensure that key features are not missed, items and response options are worded appropriately (using a precedent or intentionally deviating from it), and the resulting findings advance our understanding of comparisons and other constructs as intended. Careful consideration here will minimize participant burden while maximizing the potential benefit of new intensive assessment work on social comparison.

### Additional Points and Recommendations Emerging From This Review

As is common with scoping reviews, a few important points arose from our review of this literature that did not align precisely with our research questions. First, that studies differed in the extent to which they described providing instructions or guidelines to help participants correctly identify comparisons. As noted, two studies explicitly defined comparisons as those that were associated with psychological reactions, whereas we saw no evidence of this restriction in the remaining 31 studies. Beyond this, however, methods sections occasionally indicated that participants attended initial (baseline) meetings individually or in groups to receive instructions on recording procedures, including definitions of comparison and other constructs. The group setting of such instructions is interesting, as it raises the questions of whether participants in groups made comparisons to each other and whether such comparisons were associated with distinct reporting patterns during ambulatory assessment. These are empirical questions that, to our knowledge, have not been studied in the context of social comparison.

Theory and evidence relevant to the population of interest should guide decisions about the operational definition of social comparison for a particular study and how this information will be communicated to participants. Although the broad concept of social comparison is familiar to many potential participants, we have found in our own work that the nuances generally are not familiar; regardless of the specific definition of comparison, guidance is useful to clarify the researcher's intention and ensure high-quality responses (e.g., Arigo et al., 2019a). Further, some people believe that they do not make comparisons and/or that making comparisons is judgmental and undesirable (Hemphill and Lehman, 1991; Helgeson and Taylor, 1993). Pilot work with the population of interest is particularly helpful for identifying such beliefs, as well as appropriate language and methods for encouraging accurate responses in the natural environment and understanding the potential prevalence of comparison (or other constructs) in the population of interest (Barta et al., 2012). Although some people seem to make comparisons infrequently (Gibbons and Buunk, 1999), it is likely that instances of comparison are missed without an understanding of the range of experiences that might count. This also applies to studies that focus only on certain types of comparisons, such as upward, negative-outcome, or appearance comparisons, and extra care might be necessary to ensure that participants understand the researchers' definition. Regardless of the particular instructions given to participants, these instructions and their delivery method (e.g., in person vs. online) and setting (e.g., individual vs. group) should be clearly explained in published articles (Stone and Shiffman, 2002).

Second, a subset of studies assessed *reactivity* to social comparison recording, to determine whether the frequency of participants' comparison reports changed over the course of the recording period (e.g., Leahey and Crowther, 2008; Fardouly et al., 2017; Mills and Fuller-Tyszkiewicz, 2018). Some researchers propose that reactivity could undermine the validity of subsequent findings, as the primary construct of interest changed due to assessment rather than naturally occurring variations (see Conner and Lehman, 2012). In contrast, however, others argue that reactivity is simply an aspect of participants' learning processes. Individuals overreport many experiences in the first few days of assessment, which decreases as they adjust to the recording procedure (Iida et al., 2012). Building assessment of social comparison reactivity into intensive assessment protocols could help to clarify this process as it relates to reporting on comparison. As previous work has demonstrated that reactivity is most common when participants report on one experience exclusively (Conner and Reid, 2012), an optimal intensive assessment of social comparison might include survey items assessing other experiences in addition to comparison.

Finally, social comparisons are known to occur both effortfully (i.e., intentional seeking or generation of targets) and automatically (i.e., in response to encountering others or information about others in daily life; Gilbert et al., 1995; Suls et al., 2002), and are known to occur for different reasons (Wood, 1989). As such, it is noteworthy that only one study we reviewed assessed participants' perceptions of whether a given comparison was “deliberate or automatic” (Locke, 2005), and only one assessed participants' reason for making each comparison (Patrick et al., 2004). Because these distinctions could have important implications for the effects of comparisons in daily life and could elucidate further nuances in the comparison process, they warrant increased attention in future research.

### Strengths, Limitations, and Other Future Directions

This scoping review had several strengths, including its use of preregistered methods, adherence to PRISMA-ScR guidelines, use of a range of search terms and hand searches, and verification of correct data extraction by authors experienced in intensive assessment methods and social comparison theory. Although it is possible that relevant articles were missed, our systematic search methods make it unlikely that missed articles would meaningfully affect our conclusions or recommendations. Limitations of this review are that we did not include study findings (and as such, cannot draw conclusions about the present body of knowledge concerning specific predictors or outcomes of comparisons) or consider the consistency between conceptual definitions of comparison and items included in intensive assessments. These are valuable endeavors and deserve more attention than we could provide, within our predetermined scope (i.e., summarizing existing methods to assess comparisons within-person in the natural environment).

Consequently, essential next steps for future research are to synthesize findings from studies included in this review (as well as any relevant studies that were overlooked) and examine the overlap between conceptual definitions of social comparison and assessment items. Such syntheses could provide additional insight into relations between definitions, methods (e.g., instructions, item wording, response scales, recording frequency), and outcomes such as comparison occurrence, frequency, and consequences (e.g., for affect or behavior). A more specific focus on comparison outcomes also might facilitate synthesis of effect sizes across studies, as with meta-analysis. Lastly, although a set of concrete guidelines for conducting intensive assessment studies may be preferable to a set of considerations (see Table 4), the optimal methods depend on the specific research questions, populations, and resources researchers have at their disposal. This allows for considerable flexibility, which may better meet the needs of future work in this area.

## Conclusions

Capturing the social comparison process using intensive assessment methods has the potential to provide missing (and needed) information about how people vary in their use of and responses to comparison over short periods of time, in their natural environments. As existing work in this area has focused on the experiences of college students and on appearance comparisons among women, there is much room to expand our understanding of these processes; assessing men, older individuals, and specific groups for whom comparisons may be particularly influential (e.g., those with chronic illnesses or who undertake behavior change efforts) would be useful. In addition, given the variety of methodological options for assessing comparisons in the natural environment, there is need for greater attention to the rationales for protocol decisions and to the types of information reported in published articles. This includes descriptive information such as averages and variability estimates (with the level[s] of analysis specified), reactivity indicators, and the training participants receive to prepare them for recognizing comparisons in their daily lives. Comparisons that occur via social media, those made to imaginary targets, or those that happen automatically may be missed with intensive assessment if not specified in training or in the assessment tool itself. Further, many features of a comparison might affect its outcome, and some features that could provide needed insight rarely are assessed (e.g., identification and contrast); new studies that include assessment of these features may be particularly useful for elucidating within-person comparison processes. Finally, objectively assessed behavior represents a new frontier for understanding within-person variability in the effects of social comparison. Future work in these areas could inform both a basic understanding of comparison processes as they unfold in the real world and intervention content that responds to varying comparison needs and preferences.

## Author Contributions

DA, JM, and JS conceptualized the manuscript. MB, KP, LT, and LS completed initial data extraction. DA and JM reviewed data extraction and resolved discrepancies. All authors contributed to, reviewed, and approved the manuscript text.

### Conflict of Interest

The authors declare that the research was conducted in the absence of any commercial or financial relationships that could be construed as a potential conflict of interest.
